# Contamination Profiles of Selected Pollutants in *Procambarus clarkii* Non-Edible Portions Highlight Their Potential Exploitation Applications

**DOI:** 10.3390/jox14030049

**Published:** 2024-07-06

**Authors:** Dario Savoca, Mirella Vazzana, Vincenzo Arizza, Antonella Maccotta, Santino Orecchio, Francesco Longo, Vittoria Giudice, Gaetano D’Oca, Salvatore Messina, Federico Marrone, Manuela Mauro

**Affiliations:** 1 Department of Biological, Chemical and Pharmaceutical Sciences and Technologies (STEBICEF), University of Palermo, 90123 Palermo, Italy; mirella.vazzana@unipa.it (M.V.); vincenzo.arizza@unipa.it (V.A.); antonella.maccotta@unipa.it (A.M.); santino.orecchio@unipa.it (S.O.); francescolongo1997@gmail.com (F.L.); federico.marrone@unipa.it (F.M.); manuela.mauro01@unipa.it (M.M.); 2 NBFC, National Biodiversity Future Center, 90133 Palermo, Italy; 3 ARPA Sicilia, Agenzia Regionale Protezione Ambiente, UOC L2, via Nairobi, 90129 Palermo, Italy; vgiudice@arpa.sicilia.it (V.G.); gdoca@arpa.sicilia.it (G.D.); smessina@arpa.sicilia.it (S.M.)

**Keywords:** environmental pollution, persistent organic pollutant, trace elements, heavy metals, bioaccumulation, freshwater, invasive species, red swamp crayfish, Louisiana crawfish, sustainability

## Abstract

Properly managing aquatic organisms is crucial, including protecting endemic species and controlling invasive species. From a circular economy perspective, the sustainable use of aquatic species as a source of bioactive molecules is an area that is increasingly being explored. This includes the use of non-edible portions of seafood, which could pose considerable risks to the environment due to current methods of disposal. Therefore, it is of paramount importance to ensure that the exploitation of these resources does not result in the transfer of pollutants to the final product. This study analyzed two types of non-edible parts from the crayfish *Procambarus clarkii*: the abdominal portion of the exoskeleton (AbE) and the whole exoskeleton (WE), including the cephalothorax. These portions could potentially be utilized in the context of eradication activities regulated by local authorities. A screening analysis of four classes of pollutants, including pesticides, per- and polyfluoroalkyl substances (PFAS), phthalic acid esters (PAEs), and trace elements (TEs), was performed. The only analytes detected were TEs, and significant differences in the contamination profile were found between AbE and WE. Nevertheless, the levels recorded were comparable to or lower than those reported in the literature and below the maximum levels allowed in the current European legislation for food, suggesting that their potential use is legally permitted. In terms of scalability, the utilization of the entire non-edible *P. clarkii* portion would represent a sustainable solution for the reuse of waste products.

## 1. Introduction

The aquatic environment is undoubtedly subject to multiple stress factors, which are widely recognized as important contributors to the loss of biodiversity [1,2]. Along with habitat destruction, the invasion of alien species and environmental pollution have various negative effects on ecosystems and their individual components [3].

Among the priority pollutants that receive the most attention are trace elements (TEs), pesticides, per- and polyfluoroalkyl substances (PFAS), and phthalic acid esters (PAEs) [4,5,6,7,8,9].

These substances have been widely used for their favorable and versatile physic-chemical properties, providing numerous benefits to society. For instance, pesticides play a crucial role in modern agriculture by combating biotic factors that pose threats to crops; organometallic compounds serve as antifouling agents in ship paints; biocides have been used in polymers, textiles, etc.; TEs such as zinc and iron have been used as soil improvers; and PFAS and PAEs have been used as additives or constituents to improve the characteristics of different products, such as plastics and textiles [3,9,10,11,12].

The distribution of these pollutants in the aquatic environment is widespread, and they can accumulate and magnify in the food chain, posing risks to both wildlife and human health. In fact, TEs, pesticides, PFAS, and PAEs have been shown to be associated with a wide range of health problems, which include systemic toxicity and multifactorial diseases, such as reproductive pathologies, metabolic syndromes, teratogenicity, hepatotoxicity, cytotoxicity, oxidative stress, neurodevelopmental changes, genetic aberrations, and even carcinogenesis [10,11,13,14]. In addition, many of these chemicals are classified as endocrine disruptors due to their ability to interfere with normal endocrine activity [7,10,11,14]. The effects of exposure to contamination are dependent on the conditions of exposure, including chemical and physical parameters, as well as the presence of multiple exogenous factors that lead to co-exposure [15]. This, in turn, affects the severity of the adverse effects, which may vary depending on the type of pollutant and the level of exposure [15].

Natural and semi-natural aquatic ecosystems and cultivated areas are particularly susceptible to contamination by these pollutants, which originate from a variety of sources, including industrial effluents, agricultural practices, atmospheric deposition, and urban activities such as the dispersal of contaminated materials like plastic waste [4,7,14].

Several studies have found that aquatic organisms easily bioaccumulate these pollutants [4,5,6,7,8,11,12,14,16,17,18,19,20,21,22,23,24,25]. Among these, particular attention has recently been paid to *Procambarus clarkii* (Girard, 1852), an alien and invasive species widely recognized as a bioaccumulator of pollutants, particularly of toxic elements [16,17,18,19,20,21,22,23,24,25].

*P. clarkii*, also called Louisiana red swamp crayfish, is a cambarid decapod that is native to Northeastern Mexico and the southeast of the United States. It has been highly valued in aquaria and aquaculture due to its adaptability and prolific breeding [26,27]. However, the unintentional or deliberate release of this species into freshwater environments has resulted in its worldwide invasion, with the exception of Oceania and Antarctica, which has led to a significant impact on the native aquatic biota [27].

*P. clarkii* meat is widely appreciated, especially in China and the USA; however, due to pollutant contamination, there is a potential health risk to consumers [27,28,29,30]. There is also ongoing research into the sustainable use of other non-edible parts of decapods, such as the exoskeleton. For instance, research has shown that it is possible to extract significant amounts of chitosan from the exoskeletons of crustaceans [31]. Chitosan is an amino polysaccharide that is obtained by partially deacetylating chitin from the exoskeleton. It is a versatile natural compound that is biocompatible, biodegradable, and non-toxic, making it widely applicable in conventional pharmaceuticals as a potential excipient for formulation [32,33]. In addition, the exoskeletons of crustaceans are considered an important source of other bioactive compounds, such as minerals, carotenoids, lipids, and other amino acid derivatives. These value-added components have applications in various industries (e.g., food, nutraceutical, cosmeceutical, agro-industrial, bioplastic production, healthcare, and pharmaceutical sectors) [34,35,36]. However, different anatomical parts of crustaceans, such as the hepatopancreas and exoskeleton, have been found to have high contamination levels of different pollutants [17,22,23,37,38,39,40]. In general, waste from the seafood industry poses significant environmental and health risks. The most common disposal method is combustion, which is environmentally costly due to the low burning capacity of this waste [31].

Nowadays, from a circular economy perspective, the sustainable use of aquatic species as a source of bioactive molecules is an increasingly promising area [32,33,34,35,40].

Consequently, the analysis of pollutants’ presence and concentrations in the different parts of *P. clarkii* is of paramount importance, both to address the intended use and to determine whether the entire waste or only a portion thereof can be utilized.

In the context of sustainable valorization approaches for crustacean waste, the purpose of this study was to compare the levels of pesticides, PFAS, PAEs, and TEs in two different types of *P. clarkii* non-edible parts: the portion of the abdominal exoskeleton and the whole exoskeleton, including the cephalothoracic part. Furthermore, in this study, the concentrations registered between different individuals sampled at three Sicilian collection sites were compared in order to assess any differences in the contamination profile.

## 2. Materials and Methods

### 2.1. Sampling Campaign and Sample Preparation

**Figure 1:**
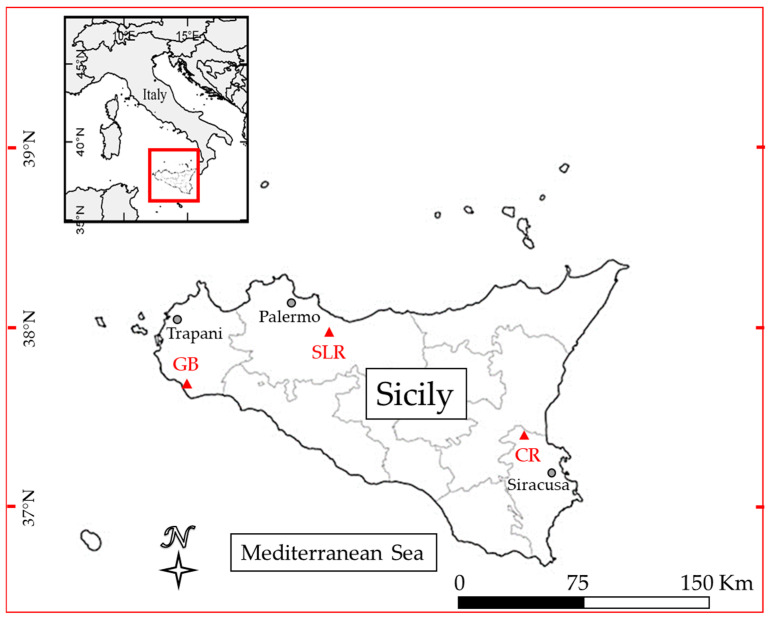
Map of sampling sites: Gorgo Basso (GB), San Leonardo River (SLR), and Cuccumella Reservoir (CR).

Based on previous distribution investigations [41,42,43,44,45], individuals of *P. clarkii* were sampled at three Sicilian sites: Gorgo Basso (GB) (Mazara del Vallo, Trapani) (coordinates: 37.609876° N, 12.654905° E), the San Leonardo River (SLR) (Caccamo, Palermo) (coordinates: 37.905663° N, 13.609423° E), and the Cuccumella Reservoir (CR) (Lentini, Siracusa) (coordinates: 37.358650° N, 14.927290° E) (Figure 1).

At these three sites, individuals of *P. clarkii* were caught using baited hoop traps, as described in Vecchioni et al. (2020), for a total of 925 individuals (159 from GB, 266 SLR, and 500 from CR). Only the amount required to form standardized sample pools for each site was randomly selected. In detail, 365 specimens were dissected: 122 from Gorgo Basso, 71 individuals from the San Leonardo River, and 87 from the Cuccumella Reservoir.

Each group was divided into six subsamples. Three groups were dissected, with only the abdominal exoskeleton (AbE) collected from each. The remaining three groups underwent a different procedure, with the whole exoskeleton (WE) being collected, including the cephalothoracic portion (without the soft tissue). Samples were pooled in order to collect the requisite quantity of exoskeleton for all of the pollutant analyses. This was performed in order to increase the homogeneity and representativeness of the measurements and to enable comparisons to be made between the different investigated analytes, which were analyzed from the same pool.

All samples were homogenized, weighed, and stored at −20 °C until freeze-drying (Alpha 2-4 LD plus freeze-dryer, Martin Christ, Osterode am Harz, D.), followed by extraction processes that differed according to the class of analytes.

The percentage of water in WE and AbE samples was determined considering the initial wet weight (w.w.) and final dry weight (d.w.) according to the equation

(1) $$\%\;H_{2}O\;in\;sample=100-\left(\frac{d.w.\;}{w.w.\;}\times 100\right)$$

The TE concentration in the wet weight was calculated to compare the concentration between Abe and WE samples considering the initial wet weight. The equation used was the following:

(2) $$w.w.=\left[TE\right]\;d.w.\;\times \left(1-\frac{\%\;H_{2}O}{100}\right)$$

### 2.2. Analysis of Exoskeleton Samples

The classes of pollutants investigated were trace elements (TEs), pesticides, per- and polyfluoroalkyl substances (PFAS), and phthalic acid esters (PAEs).

The analytical methods/protocols and techniques used for the determination of TEs in the *P. clarkii* exoskeleton are reported in the Supplementary Materials.

The extraction and analyses of pesticides, PFAS, and PAEs were conducted by “Chimica Applicata Depurazione Acque s.n.c. di Filippo Giglio e C” (CADA). The methods/protocols used were UNI EN 15662:2018 for pesticides, MPI 268 2023 Rev.0 for PFAS, and EPA 3541 1994 + EPA 3620C 2014 + EPA 8270E 2018 for PAEs.

For trace element extraction and analysis, the procedures were performed according to Savoca et al. [6]. Briefly, about 0.6 g of each sample was digested in a solution mixture containing 8 mL HNO_3_ (67–69.0%, Carlo Erba Ultrapure for trace analysis) and 2 mL H_2_O_2_ (H_2_O_2_ 30.0%, for trace analysis, Sigma Aldrich, Milan, Italy) in Teflon containers using a microwave digestion system (Ethos Up Milestone Connect High-Performance Microwave Digestion System) operated from 20 °C to 180 °C in 15 min, maintained at 180 °C for 55 min, and cooled for 10 min. Then, the samples were diluted to a volume of 20 mL with double-distilled water, filtered with syringes equipped with cellulose acetate filters (pore size: 0.45 µm, diameter: 25 mm, AISIMÔ CORPORATION CO., LTD, London, UK), and analyzed through inductively coupled plasma mass spectrometry (Agilent 7800 ICP-MS with SPS4 autosampler Agilent Technologies, Milan, Italy).

The analytical procedure used was in accordance with the EPA 200.8 method supplemented by EPA 6020b and three replicates were performed for each measurement. The procedures to verify possible contamination in the measurement system and the description of the multielement calibration process are reported in Savoca et al. [6].

The calibration range used was 0–160 ppb for all elements except mercury (range of 0 to 4 ppb) and the correlation coefficient (R2) was = 0.9999 for each element.

A parallel analysis of procedural blanks and certified reference material (CRM: NIST-1566b (Oyster Tissue) and DORM-4 (Fish Protein)) enabled quality checks and accuracy verification. Such analyses showed a less than 10% relative standard deviation.

The limit of detection (LOD) and limit of quantification (LOQ) were calculated as the concentration equivalent to the mean (10 repeated blank measurements) of the selected analytical mass signals +3 (LOD) or +10 (LOQ) the standard deviation (SD). The complete list of all of the analytes and other available information on the percentages of recovery and limits of detection and quantification is reported in the Supplementary Materials (Tables S1 and S2).

### 2.3. Statistical Analysis

To assess the significance of the differences in the contamination profiles between sites and sample types (AbE vs. WE), a permutational multivariate analysis of variance (PERMANOVA) was performed. The experimental design consisted of two factors, namely the site (two levels, fixed and orthogonal) and sample type (two levels, fixed and orthogonal), and 15 variables corresponding to the detected trace elements. Each term in the analysis was tested using 999 random permutations based on the Euclidian distance and normalized dataset. For the statistical analysis, if the elemental concentration values were not detected (i.e., below the limit of detection, LOD), they were substituted with the corresponding LOD/2. PERMANOVA analyses were performed using the PAST software 4.04 [46]. The distribution of the trace element concentrations in all samples was graphically represented by a box and jitter plot, where the 25–75th percentiles were drawn using a box, the minimum and maximum were shown at the ends of the thin lines (whiskers), and the median was marked as a horizontal line in the box.

## 3. Results

**Figure 2:**
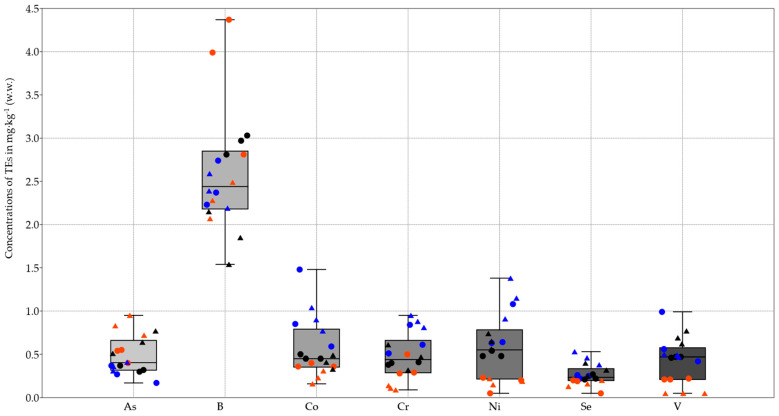
The box and plot show the concentrations of TEs expressed in mg·kg^−1^ wet weight (w.w.) found in two types of exoskeleton samples: the whole exoskeleton (WE), represented by a triangle, and the abdominal exoskeleton portion (AbE), represented by a dot. The 25th to 75th percentiles are indicated by a box. The minimum and maximum values are shown at the ends of the thin lines (whiskers). The median is marked as a horizontal line in the box plot. The site of the samples is differentiated by color: red for Gorgo Basso (GB), blue for the San Leonardo River (SLR), and black for the Cuccumella Reservoir (CR). For Ni, Se, and V, the minimum values correspond to the LOD/2 of 0.05 mg·kg^−1^.

**Figure 3:**
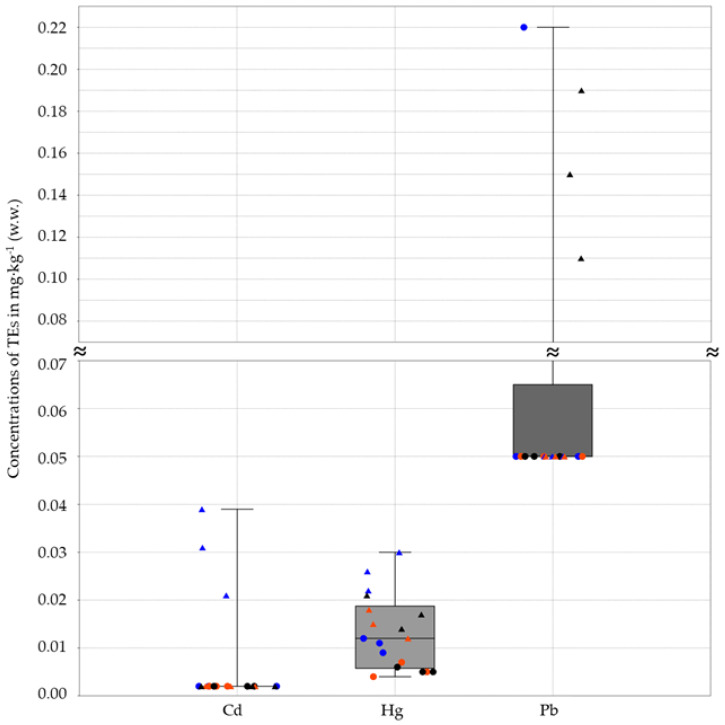
The box and plot show the concentrations of TEs expressed in mg·kg^−1^ wet weight (w.w.) found in two types of exoskeleton samples: the whole exoskeleton (WE), represented by a triangle, and the abdominal exoskeleton portion (AbE), represented by a dot. The 25th to 75th percentiles are indicated by a box. The minimum and maximum values are shown at the ends of the thin lines (whiskers). The median is marked as a horizontal line in the box plot. The site of the samples is differentiated by color: red for Gorgo Basso (GB), blue for the San Leonardo River (SLR), and black for the Cuccumella Reservoir (CR). For Cd and Pb, the minimum values correspond to the LOD/2 of 0.002 mg·kg^−1^ for Cd and 0.05 mg·kg^−1^ for Pb.

**Figure 4:**
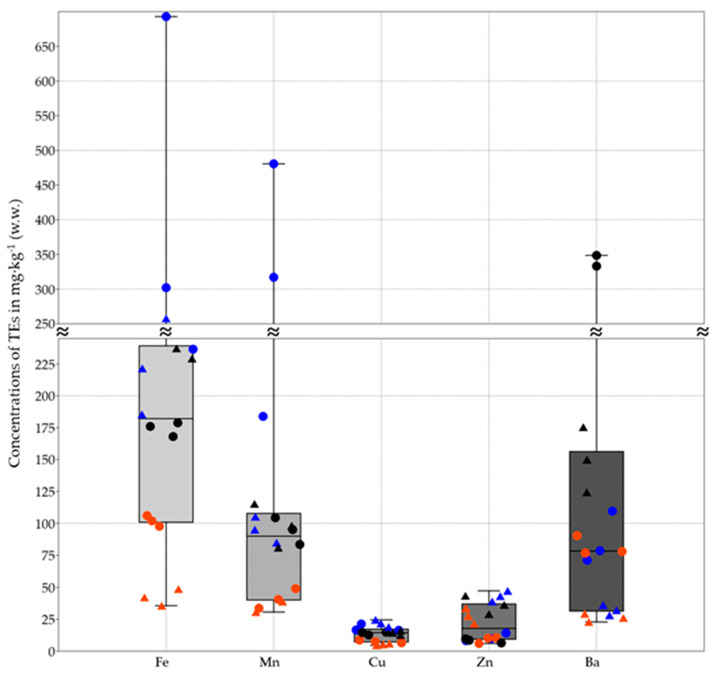
The box and plot show the concentrations of TEs expressed in mg·kg^−1^ wet weight (w.w.) found in two types of exoskeleton samples: the whole exoskeleton (WE), represented by a triangle, and the abdominal exoskeleton portion (AbE), represented by a dot. The 25th to 75th percentiles are indicated by a box. The minimum and maximum values are shown at the ends of the thin lines (whiskers). The median is marked as a horizontal line in the box plot. The site of the samples is differentiated by color: red for Gorgo Basso (GB), blue for the San Leonardo River (SLR), and black for the Cuccumella Reservoir (CR).

The analysis of the PAEs, PFAS, and pesticides showed results below the limit of quantification for all samples. Differently, except for antimony, tin, and silver, which were not detected, the TE analysis showed different concentrations between the two exoskeleton sample types (AbE and WE) (Figure 2, Figure 3 and Figure 4).

The mean concentration levels of the trace elements of the different samples analyzed are given in Table 1.

On average, for WE, the elemental concentrations of the elements were in the following descending order: Fe > Mn > Ba > Zn > Cu > B > Ni > As > Co > Cr > V > Se > Pb > Hg > Cd. Meanwhile, Sb, Ag, and Sn were below the detection limit. For AbE, the decreasing order of the concentrations of TEs was as follows: Fe > Ba > Mn > Cu > Zn > B > Co > Ni > Cr > V > As > Se > Pb > Hg. Meanwhile, Sb, Ag, Sn, and Cd were below the concentration limit.

Comparing the TE concentrations found in Abe and WE between the same sampling site, with the exception of some cases (Fe, V, and Mn for SLR and GB; Pb for SLR; Cr for GB; and Ba and Co for the three sites), the TE levels in WE were higher than those in AbE (Figure 2, Figure 3 and Figure 4).

Additionally, higher concentration ratios of WE/AbE were observed for Hg (2.44 in SLR, 2.77 in GB, and 3.33 in CR) and Zn (4.01 in SLR, 3.10 in GB, and 4.43 in CR), while, for Ba, the concentration was higher in AbE than WE, with a ratio of 2.69 in SLR, 3.13 in GB, and 2.07 in CR.

Moreover, considering only the two types of samples, on average, the concentrations of As, Cd, Cr, Cu, Hg, Ni, Pb, Se, and Zn were higher in WE than in Abe, while B, Co, Ba, Mn, and V were more concentrated in Abe than in WE. On average, the levels of Hg and Zn were 2.74 and 3.84 times higher in WE than in AbE, respectively, while, on average, the concentration of Ba was 2.30 times higher in AbE than in WE.

Overall, considering the 15 TEs detected, significant differences between the concentration levels of all elements were found between the three sites (*p* = 0.0001) and the different types of exoskeletal samples (*p* = 0.0001), as well as in the interaction of the latter two factors (*p* = 0.0001).

With regard to site contamination, both types of samples from GB had a higher concentration of As, whereas the Ba concentrations were significantly higher in samples from CR (Figure 1). Finally, SLR samples were found to be more contaminated for Co, Cr, Hg, Ni, Cu, Se, and Zn (Figure 2, Figure 3 and Figure 4).

## 4. Discussion

Along with the parastacid *Cherax destructor* (Clark, 1936) and the portunid *Callinectes sapidus* (Rathbun, 1896), *P. clarkii* is among the few non-native decapod crustaceans occurring in Sicilian inland waters [41,42,43]. After its initial introduction in the early XXI century, the Louisiana red swamp crayfish was observed in several different hydrographical basins, thus suggesting multiple, independent release events of the species in the wild [44]. Nowadays, *P. clarkii* is widespread in the permanent water bodies of the island [41], whereas it has never been observed in temporary swamps, ponds, or streams [45].

In the context of this survey, and considering their ability to bioaccumulate pollutants, individuals of *P. clarkii* were sampled in three Sicilian sites and their exoskeletons analyzed for the presence of PFAS, pesticides, PAEs, and trace elements.

The sole pollutants identified in this study were trace elements. It is crucial to acknowledge that chitin is a metal-chelating polymer. Nevertheless, it is probable that animals from contaminated regions exhibit a higher proportion of metal-polluting ions in their exoskeletons [47]. The lower the contamination in the exoskeleton, the greater the ability of the exoskeleton to chelate metal ions. The analysis of trace elements in the exoskeleton therefore indicates both the state of environmental contamination by these pollutants and the possibility of the exploitation of this matrix for different applications, such as in the metal bioremediation of wastewater [48].

Non-essential elements, such as Hg, Pb, and Cr, are regarded as xenobiotic and are toxic even at low concentration levels, while essential elements, such as Fe, Cu, and Zn, may cause toxic effects in organisms when their concentrations exceed certain values [6]. In this context, it is important to note that the chemical properties of elements may vary considerably based on the different ligand and/or valence states, affecting their absorption behavior and toxicity [22]. Hexavalent chromium and methylmercury, for instance, are among the non-essential elements of greatest concern [19,22]. Methylmercury also represents approximately 90% of the total mercury present in crayfish [19].

In general, the TE levels found are dependent on both the environmental pollution and the different organotropism of the elements, namely the distinct biodistribution of the elements in the various anatomical parts of the organism [49]. The analyses revealed that the abdominal portion of the exoskeleton exhibited higher Ba concentrations than the entire exoskeleton. In contrast, the entire exoskeleton exhibited higher levels of Hg and Zn than the abdominal portion alone, indicating that these elements are more present in the cephalothorax. Although the exoskeleton/carapace includes all of the mineralized structure that covers and protects the entire decapod crustacean [50], in most studies conducted, only the abdominal portion [22,24] or exoskeleton and gills were analyzed [51]. In some cases, the specific portion analyzed was not specified [20,21].

Comparing our results with those of other studies, the concentrations that we found are similar or lower [20,21,22,23,24], except for the levels of Ba, which could only be compared to one study [22].

From four sampling sites in the River Nile, in Egypt, Shaaban et al. [21] evaluated the seasonal variations in the Cd and Pb levels in different tissue types of *P. clarkii*, including the exoskeleton. The latter was found to have the highest concentrations of these metals during the summer. Very high Cd and Pb levels in the exoskeletons collected from four sites were found, namely 1.49 mg·kg^−1^ d.w. and 12.25 mg·kg^−1^ d.w., respectively. These concentration values were also significantly higher than our observations (see Table 1 and Equation (2)). Xiong et al. [22] analyzed several TEs in the exoskeleton of *P. clarkii* in natural and cultivated areas. The mean levels recorded in the natural environment were reported in the wet weight and were 15.2 mg·kg^−1^ for Cu, 2.43 mg·kg^−1^ for Pb, 18.1 mg·kg^−1^ for Zn, 605 mg·kg^−1^ for Fe, 131 mg·kg^−1^ for Mn, 1.18 mg·kg^−1^ for Cd, 6.25 mg·kg^−1^ for Cr, 20.7 mg·kg^−1^ for Ba, and 0.59 mg·kg^−1^ for As. Meanwhile, in the cultivated environment, they were the following: 15.7 mg·kg^−1^ for Cu, 1.27 mg·kg^−1^ for Pb, 14.4 mg·kg^−1^ for Zn, 272 mg·kg^−1^ for Fe, 146 mg·kg^−1^ for Mn, 0.45 mg·kg^−1^ for Cd, 13.04 mg·kg^−1^ for Cr, 17.2 mg·kg^−1^ for Ba, and 0.57 mg·kg^−1^ for As. The average concentrations of TEs in crayfish in cultivation were generally lower than those of crayfish in the wild statistical analysis, which showed that the main sources were agricultural activities and vehicular emissions [22]. Although the levels of Cu, Zn, Mn, and As were similar to those in our study, we observed much lower levels of Pb, Fe, Cd, and Cr than Xiong et al. [22].

In contrast, our findings revealed higher Ba levels than those previously reported in China [22]. In this latter study, the greatest Ba concentration was observed in the exoskeleton and sediments [22].

In addition to proteins and carotenoids (mainly astaxanthin and its esters), the chiton-based exoskeletons of crustaceans are composed of mineral salts, primarily calcium carbonate (as a reinforcing component) [35,50]. Given the vicariance between barium and calcium [49], it is plausible that the exoskeleton accumulates substantial quantities of Ba due to the direct contact with sediments [20]. Ba is a non-essential element with relatively low toxicity; however, elevated levels are a cause for concern [6]. In particular, although the consumption of animals is often restricted to the muscle tissue, such concern may arise in cases where individuals of *P. clarkii* are eaten whole by other predators or, in some cases, by humans [52]. The different levels of elemental concentrations in environmental matrices often reflect the levels of contamination found in the biota living in such environments. The concentration levels of different elements in biota samples are indicative of the degree of contamination of the environmental matrices [51]. Gedik et al. [20] also detected similar levels of Cd, Cu, and As in their study. However, their study showed higher levels of Pb compared to our findings. In contrast, we found higher levels of Zn in samples containing the cephalothorax portion, suggesting that Zn is more present in this part than in the abdominal carapace portion.

This result is consistent with other studies on the bioaccumulation of Zn in freshwater crustaceans, which have demonstrated higher concentrations of this element in the cephalothoracic portion than in the abdominal portion [53].

Abd El-Aziz et al. [24] found higher levels of Co, Pb, and Cd at two sites in Egypt, while Zn, Fe, and As were of the same order of magnitude.

Comparing the elemental concentrations with those obtained by Marić and Stanić [23] in another freshwater decapod (*Astacus astacus* (Linnaeus, 1758)), we found similar concentrations of trace elements (TE) in chitin for some essential elements (Cu, Zn), except for Fe and Mn, which showed higher levels in our study. In addition, *P. clarkii* had Ni and Cr concentrations that were an order of magnitude higher than in *A. astacus*, although the As and Hg concentrations in *A. astacus* were two and four orders of magnitude higher than in *P. clarkii*, respectively. Regarding the maximum levels allowed by the European legislation, Commission Regulation (EU) 2023/915 of 25 April 2023, on the maximum levels of certain contaminants in food and feed, sets the TE concentration limit for crustaceans of 0.5 mg·kg^−1^ w.w. for cadmium, lead, and mercury [54]. This maximum level applies only to muscle meat from appendages and the abdomen and does not include the exoskeleton component. Our results are significantly lower than these levels. Additionally, the exoskeleton could serve as a source of chitin and chitosan, which can be used as functional additives in food, industrial feed, and fertilizers in agriculture. However, the quantities of the exoskeletal matrix required for the final product would only constitute a small percentage. In this sense, the amount or percentage of this matrix in the final product would be lower and would comply with the threshold limits.

Similarly, the levels found are also acceptable for other trace elements; in fact, although no EU limit for the human consumption of crayfish muscle has been established, other national regulations set maximum limits, e.g., Cu to 20 mg·kg^−1^ w.w. in edible mass for Zn to 70 mg·kg^−1^ w.w. [23]. In addition, the maximum permitted limits for heavy metals in food are lower than those established for cosmetics, potentially allowing the use of exoskeletons in this sector [55].

In our research study, we conducted sampling during the summer, when the highest TE concentrations are typically recorded [21]. It is suggested that removing *P. clarkii* individuals would be even more beneficial in other seasons for the ideal exploitation of this resource.

Concerning organic pollutants (PFAS, PAEs, and pesticides), the results indicated levels below the detection limit, suggesting either that the sampling sites were not contaminated by these substances or that these lipophilic pollutants were not particularly similar to the investigated exoskeletal matrix.

In general, few studies concern the bioaccumulation of organic pollutants in *P. clarkii* and focus on edible portions, soft tissue, or the whole body [25,56,57]. The low contamination (or the contamination below the detection limit) found in our work is a noteworthy result. Standard industrial practices for chitin extraction involve washing processes, such as boiling in acetone and washing in hot ethanol, to remove any residual contaminants. In light of the above, these steps could be avoided [36]. In any case, even based on the intended use, continuous biomonitoring over time for the various classes of contaminants at sites of interest is essential.

The study’s findings highlight significant differences in the TE contamination profiles between the two types of non-edible portions of *P. clarkii* and across the three different sites. It is worth noting that the contamination levels detected in both WE and AbE are relatively low when compared to previous research and regulatory standards. Consequently, it is crucial to consider these differences when undertaking processes for the transformation of the waste portions of seafood. This is particularly important due to the presence of bioaccumulating components, such as the hepatopancreas in the cephalothorax, which could facilitate their transfer to the final product. By considering these factors, it is possible to ensure the production of seafood waste for reuse that meets the necessary safety and quality standards.

In general, the marketing of *P. clarkii* is limited in several countries; however, its exploitation would be possible in relation to particular cases. As part of the control, management, and eradication activities of *P. clarkii* required by the EU and Italian legislation [39], the use of removed specimens resulting from these activities could be allowed, making the actions economically more sustainable for the local managing authorities. This would avoid the carcasses being considered as waste and having to be disposed of at an additional cost and would also help to cover the costs of managing this invasive species, thus generating positive economic and environmental impacts. In fact, the most common disposal method is combustion, which is environmentally costly due to the low burning capacity of this waste [31]. In this way, the edible portion of the individuals could be used for food or feeding purposes, while the waste, such as the exoskeleton, could be repurposed, such as for the production of animal feed, for the extraction of molecules of biotechnological interest, or for nutraceutical, cosmetic, or pharmaceutical/biomedical purposes, as well as other uses, such as in biorefineries [32,33,34,35,40]. In general, waste from the seafood industry poses significant environmental and health risks.

A comparison of the contamination profiles of the two types of exoskeleton portions may be useful for future research on waste from the seafood processing industry, such as the use of the exoskeleton to isolate chitosan or to understand whether there are pollutants and any differences in contamination between different types of seafood waste. In this context, it is important to emphasize that the production of seafood waste should be quick, economic, sustainable, and safe from the perspective of contamination to enable the reuse of waste biomass. Consequently, using the entire portion of the exoskeleton/carapace, which is more quickly obtainable than the more laborious dissection of the abdominal portion, represents the preferable option as it is more scalable. Within this framework, the European Commission promotes policies and regulations that aim to endorse environmental sustainability. One such action is the eradication of *P. clarkii*, which is particularly important for the most threatened ecosystems. Therefore, the regulated eradication of Louisiana crayfish could improve ecosystem health and support scientific and biotechnological progress, as well as profiting from the use of different parts of the decapod. After analysis, the muscle portion of *P. clarkii* may be suitable for food or feed; meanwhile, the exoskeleton, which is typically discarded, could be a valuable resource in various industries, such as food additives, nutraceuticals, cosmetics, and biomedicine, after proper processing.

## 5. Conclusions

This study found no significant contamination by PFAS, phthalates, or pesticides. However, trace elements, including heavy metals, were detected. Specifically, the analysis of trace elements showed significant differences in the contamination levels between the two types of exoskeleton samples, suggesting that the cephalothoracic portion contributes to these differences in contamination. In addition, the sampling areas have an impact on the contamination of the exoskeletons of different individuals. Therefore, it is recommended to systematically carry out chemical analyses for both biomonitoring activities and to assess the contamination of the exoskeletal matrix before using it for different potential target sectors. Comparisons with other studies showed that the contamination levels in our samples were similar or lower.

It can be reasonably inferred from the evidence that the cephalothoracic portion of *P. clarkii* contributes to the different contamination profile of trace elements compared to the exoskeletal abdominal portion alone. This suggests that both portions could potentially be exploited. Therefore, given that the production and reuse of the entire exoskeleton could be a faster and more scalable operation, this work provides a starting point for future research and applications.

## Figures and Tables

**Table 1:** Mean concentration levels of trace elements (mg·kg^−1^ wet weight (w.w.) based on 65% of water for WE and 40% for AbE ± standard deviation (SD), analyzed in exoskeleton pools with (WE) or without (AbE) cephalothorax portions of *P. clarkii* collected from Gorgo Basso (GB), San Leonardo River (SLR), and Cuccumella Reservoir (CR). Sb, Ag, and Sn were below the detection limit for all samples analyzed.

Matrix	Site	As	B	Cd	Co	Cr	Fe	Mn	Hg	Ni	Pb	Cu	Se	V	Zn	Ba
WE (n = 3)	GB	0.83 ± 0.11	2.28 ± 0.21	<	0.23 ± 0.08	0.12 ± 0.03	42 ± 7	33 ± 4	0.015 ± 0.003	0.19 ± 0.04	<	5.2 ± 0.7	0.16 ± 0.04	<	27.6 ± 6.0	26 ± 3
WE (n = 3)	SLR	0.36 ± 0.05	2.39 ± 0.20	0.030 ± 0.009	0.90 ± 0.14	0.88 ± 0.07	222 ± 36	95 ± 10	0.026 ± 0.004	1.15 ± 0.24	<	21.8 ± 2.8	0.46 ± 0.08	0.49 ± 0.02	43.1 ± 4.1	32 ± 4
WE (n = 3)	CR	0.64 ± 0.13	1.85 ± 0.31	<	0.41 ± 0.08	0.47 ± 0.15	237 ± 8	98 ± 17	0.017 ± 0.004	0.65 ± 0.09	0.15 ± 0.04	14.3 ± 1.7	0.32 ± 0.08	0.69 ± 0.07	36.3 ± 7.2	150 ± 26
AbE (n = 3)	GB	0.50 ± 0.08	3.72 ± 0.81	<	0.37 ± 0.03	0.36 ± 0.13	102 ± 4	41 ± 8	0.005 ± 0.002	0.16 ± 0.10	<	7.6 ± 1.1	0.14 ± 0.08	0.22 ± 0.004	8.9 ± 2.5	82 ± 8
AbE (n = 3)	SLR	0.27 ± 0.10	2.45 ± 0.26	<	0.97 ± 0.46	0.65 ± 0.17	411 ± 247	327 ± 149	0.011 ± 0.002	0.78 ± 0.26	0.11 ± 0.10	18.1 ± 2.8	0.24 ± 0.02	0.65 ± 0.30	10.7 ± 3.1	86 ± 20
AbE (n = 3)	CR	0.33 ± 0.04	2.93 ± 0.11	<	0.47 ± 0.03	0.40 ± 0.01	174 ± 6	94 ± 10	0.005 ± 0.001	0.50 ± 0.04	<	14.0 ± 1.2	0.23 ± 0.03	0.47 ± 0.01	8.2 ± 1.6	310 ± 54

n: number of pools; <: below the quantification limit.

## Data Availability

The data presented in this study are available in this article and Supplementary Materials.

## Supplementary Materials

The following supporting information can be downloaded at: https://www.mdpi.com/article/10.3390/jox14030049/s1, Table S1: List of trace element and related LOD, LOQ and recovery percentage obtained by analysis of CRM; Table S2: List of pesticides, PFAS and PAEs and their limit of detection (LOD) expressed in mg kg^−1^.
