# Supplementary material for: Contamination Profiles of Selected Pollutants in Procambarus clarkii Non-Edible Portions Highlight Their Potential Exploitation Applications
Source: J Xenobiot. 2024 Jul 6;14(3):893–906. doi: 10.3390/jox14030049 (PMC11270285; doi:10.3390/jox14030049)
Supplement: Supplementary file 1 [file jox-14-00049-s001.zip › jox-3023884-supplementary.pdf]

Supplementary Materials

Table S1 List of trace element and related LOD, LOQ and recovery percentage obtained by analysis of CRM.

**Element**	**R_{CS}%**	**LOD_{CS} (µg g^{-1})**	**LOQ_{CS} (µg g^{-1})**
Antimony (Sb)	118	0.10	0.33
Arsenic (As)	85	0.10	0.33
Boron (B)	84	0.10	0.33
Cadmium (Cd)	88	0.005	0.02
Cobalt (Co)	91	0.10	0.33
Chromium (Cr)	99	0.05	0.16
Iron (Fe)	85	1.34	4.56
Manganese (Mn)	85	0.29	0.98
Mercury (Hg)	85	0.001	0.003
Nickel (Ni)	117	0.10	0.33
Lead (Pb)	92	0.10	0.33
Copper (Cu)	92	0.10	0.33
Selenium (Se)	85	0.10	0.33
Vanadium (V)	117	0.10	0.33
Zinc (Zn)	85	0.57	1.96
Silver (Ag)	98	0.10	0.33
Tin (Sn)	105	0.10	0.33
Barium (Ba)	96	0.10	0.33

R_{CS} recovery percentage of crayfish samples; **LOD_{CS}**: limit of detection crayfish samples; **LOQ_{CS}**: limit of quantification crayfish samples.

Table S2. List of pesticides, PFAS and PAEs and their limit of detection (LOD) expressed in mg kg^{-1}

**Pesticides**	**LOD**
2,4-DDD	0.005
2,4-DDE	0.005
2,4-DDT	0.005
4,4-DDD	0.005
4,4-DDE	0.005
4,4-DDT	0.005
Alachlor	0.005
Acephate	0.005
Aldrin	0.005
α-Chlordane	0.005
α-Hexachlorocyclohexane	0.005
Atrazine	0.005
Azinphos-ethyl	0.005
Azinphos-methyl	0.005
β-Hexachlorocyclohexane	0.005
Bolstar (Sulprofos)	0.005
Bromophos-ethyl	0.005
Bromophos-methyl	0.005
Butachlor	0.005
Chlormephos	0.005
Clordecone	0.005
Clorfenvinphos (mixture of Z and E isomers)	0.005
Chlorobenzilate	0.005
Cloroneb	0.005

Chlorothalonil	0.005
Chlorpyrifos-methyl	0.005
Chlorpyrifos-ethyl	0.005
Coumaphos	0.005
Dactal	0.005
Demeton-O	0.005
Demeton-S	0.005
Demeton-S-metile	0.005
Diazinon	0.005
Dichlorvos	0.005
Dieldrin	0.005
Dimethoate	0.005
Endosulfan Sulfato	0.005
α-Endosulfan	0.005
β-Endosulfan	0.005
Endrin	0.005
Endrin aldehyde	0.005
Heptachlor	0.005
Heptachlor epoxide	0.005
Heptenophos	0.005
Hexachlorobenzene (HCB)	0.005
Ethoprophos	0.005
Fenarimol	0.005
Fenclorfos	0.005
Fenitrothion	0.005
Fensulfothion	0.005
Fenthion (fention and its oxygenated analogue, their sulfoxides and sulfones)	0.005

Fonofos	0.005
Formothion	0.005
γ-Chlordane	0.005
γ - hexachlorocyclohexane (Lindane)	0.005
Iodofenphos	0.005
Isodrin	0.005
Isofenphos	0.005
Malathion	0.005
Merphos	0.005
Methidathion	0.005
Metolachlor	0.005
Methoxychlor	0.005
Mevinphos	0.005
Mirex	0.005
Naled (dibrom)	0.005
Trans-Nonachlor	0.005
Cis- Nonachlor	0.005
Norflurazon	0.005
Paraoxon-ethyl	0.005
Paraoxon-methyl	0.005
Parathion methyl	0.005
Parathion ethyl	0.005
Pentachlorobenzene	0.005
Permethrin (cis)	0.005
Permethrin (trans)	0.005
Phorate	0.005
Phorate oxon	0.005
Phosalone	0.005

Phosphamidon	0.005
Piridafention	0.005
Pirimiphos-Ethyl	0.005
Prometryn	0.005
Pronamide	0.005
Propachlor	0.005
Quinalphos	0.005
Simetryn	0.005
Sulfotepp	0.005
Terbacil	0.005
Tetrachlorvinphos	0.005
Tokuthion	0.005
Triadimefon	0.005
Triazophos	0.005
Trichlorfon	0.005
Trichloronate	0.005
**PFAS**	**LOD**
PFBA (Perfluorobutanoic acid)	0.002
PFBS (Perfluorobutanesulfonic acid)	0.002
PFPeA (Perfluoro-n-pentanoic acid)	0.002
PFHxA (Perfluorohexanoic acid)	0.002
PFHxS (Perfluorohexanesulfonic acid)	0.002
PFHpA (Perfluoro-n-heptanoic acid)	0.002
PFOA (Perfluorooctanoic acid)	0.002
PFNA (Perfluorononanoic acid)	0.002
PFDA (Perfluorodecanoic acid)	0.002
PFODA (Perfluoro-n-octadecanoic acid)	0.002

PFUnDA (Perfluoroundecanoic acid)	0.002
**Phthalic acid esters**	**LOD**
DEHP (di(2-ethylhexyl) phthalate)	0.1
BBzP (benzyl butyl phthalate)	0.1
DEP (diethyl phthalate)	0.1
DMP (dimethyl phthalate)	0.1
DnOP (di-n-octyl phthalate)	0.1
DnHP (dihexyl phthalate)	0.1
DnBP (di-n-butyl phthalate)	0.1
DPP (di-iso-pentyl phthalate)	0.1
DMEP (bis(2-methoxyethyl) phthalate)	0.1
DnPP (di-n-pentyl phthalate)	0.1
DCHP (dicyclohexyl phthalate)	0.1
